# Evaluating Factors Affecting Knowledge Sharing Among Health Care Professionals in the Medical Imaging Departments of 2 Cancer Centers: Concurrent Mixed Methods Study

**DOI:** 10.2196/53780

**Published:** 2024-11-13

**Authors:** Maryam Almashmoum, James Cunningham, John Ainsworth

**Affiliations:** 1 Division of Informatics Imaging and Data Sciences, School of Health Sciences Faculty of Biology, Medicine, and Health The University of Manchester Manchester United Kingdom; 2 Nuclear Medicine Department Faisal Sultan Bin Eissa Kuwait Cancer Control Center Kuwait City Kuwait

**Keywords:** knowledge management, knowledge sharing, medical imaging departments, cancer centers, The Christie, Kuwait Cancer Control Center, concurrent mixed methods, factors, challenges, definition, mechanisms, practices

## Abstract

**Background:**

Knowledge sharing is a crucial part of any knowledge management implementation. It refers to sharing skills and experience among team members in an organization. In a health care setting, sharing knowledge, whether tacit or explicit, is important and can lead to better health care services. In medical imaging departments, knowledge sharing can be of particular importance. There are several factors that affect knowledge-sharing practices in medical imaging departments: individual, departmental, and technological. Evaluating the importance of these factors and understanding their use can help with improving knowledge-sharing practices in medical imaging departments.

**Objective:**

We aimed to assess the level of motivation, identify current knowledge-sharing tools, and evaluate factors affecting knowledge sharing in the medical imaging departments of 2 cancer centers, The Christie, United Kingdom, and the Kuwait Cancer Control Center (KCCC).

**Methods:**

A concurrent mixed methods study was conducted through nonprobability sampling techniques between February 1, 2023, and July 30, 2023. Semistructured interviews were used to validate the results of the quantitative analysis. Data were collected using an electronic questionnaire that was distributed among health care professionals in both cancer centers using Qualtrics. Semistructured interviews were conducted online using Microsoft Teams. The quantitative data were analyzed using the Qualtrics MX software to report the results for each question, whereas the qualitative data were analyzed using a thematic approach with codes classified through NVivo.

**Results:**

In total, 56 respondents from the KCCC and 29 from The Christie participated, with a 100% response rate (56/56, 100% and 29/29, 100%, respectively) based on the Qualtrics survey tool. A total of 59% (17/29) of health care professionals from The Christie shared their knowledge using emails and face-to-face communication as their main tools on a daily basis, and 57% (32/56) of health care professionals from the KCCC used face-to-face communication for knowledge sharing. The mean Likert-scale score of all the components that assessed the factors that affected knowledge-sharing behaviors fell between “somewhat agree” and “strongly agree” in both centers, excepting extrinsic motivation, which was rated as “neither agree nor disagree.” This was similar to the results related to incentives. It was shown that 52% (15/29) of health care professionals at The Christie had no incentives to encourage knowledge-sharing practices. Therefore, establishing clear policies to manage incentives is important to increase knowledge-sharing practices.

**Conclusions:**

This study offered an evaluation of factors that affect knowledge sharing in 2 cancer centers. Most health care professionals were aware of the importance of knowledge-sharing practices in enhancing health care services. Several challenges were identified, such as time constraints, a lack of staff, and the language barrier, which limit knowledge-sharing practices. Therefore, establishing a clear policy for knowledge sharing is vital to practicing knowledge-sharing behaviors and facing any challenges that limit this practice.

## Introduction

### Background

Knowledge consists of a combination of facts, ideas, experiences, and information that is gained through experience and practice [1]. Knowledge management is the organizational capability to identify, transfer, convert, and share knowledge to attain institutional success. In health care, knowledge is an important asset in following the best medical practices. In recent years, health care intuitions have had a clear mission to establish a strong knowledge management system to use their knowledge in a good way by sharing health care professionals’ knowledge with others [2]. Knowledge management is directly related to good performance [3]. The primary aim of knowledge management in a health care setting is to create a culture of knowledge sharing among health care professionals, allowing them to carry out hospital tasks in an efficient way, leading to an increase in successful patient outcomes and a reduction in repetitive medical errors [4,5]. Knowledge management consists of 4 processing steps: knowledge creation, knowledge capture, knowledge sharing, and knowledge application [6]. According to a 2016 report by the World Health Organization, there are many challenges that health care institutions face, such as long wait times for patient services, a lack of health care professionals, and inadequate information and communications technology (ICT) infrastructures in health care centers [1,7]. Good knowledge management practice starts with understanding each processing step and trying to identify the challenges and solutions for each of them [5,6].

Knowledge sharing in health care institutions has a positive impact on institutional performance [8]. Effective knowledge sharing has several benefits related to increasing successful patient outcomes, such as innovation, critical thinking, problem-solving, reducing medical errors, avoiding repetitive medical errors, increasing performance, and gaining competitive advantages [6,9-11]. Furthermore, improving knowledge-sharing practices among health care professionals allows them to learn and use resources efficiently [7]. Health care institutions have complex organizational structures [5]. They employ a variety of multidisciplinary professionals who communicate and share knowledge among each other as a part of day-to-day medical practice. Effective knowledge-sharing practices among health care professionals contribute to a positive overall knowledge-sharing culture [5]. Ipe [12] defined knowledge sharing as “the act of making knowledge available to others within the organizations.” Knowledge sharing is the way in which health care professionals share their tacit and explicit knowledge [13]. Tacit knowledge is defined as any thoughts and ideas that exist in the human mind [14]. It is difficult to capture and can be shared through interaction with others [15]. Explicit knowledge, in contrast, exits in the documents, policies, and manuals that departments have, and it is easy to capture [16].

Medical imaging departments consist of several divisions: radiology, nuclear medicine, radiotherapy, and physical radiation. The names of the divisions and their number differ from one hospital to another. Medical imaging departments can be considered the backbone of a hospital due to the importance of the tasks that are performed there by specialized health care professionals [17,18]. The number of health care professionals working in medical imaging departments has increased in recent years due to the expansion of the duties carried out in these departments [19].

### Factors Affecting Knowledge Sharing

There are several factors that affect knowledge-sharing practices [20-31]. These factors can differ from one institution to another depending on the nature of each environment [20-31]. Technological factors often differ between institutions due to the use of specialized technologies (eg, the use of a picture archiving and communication system [PACS] in medical imaging departments), whereas individual factors are often the same across institutions as they relate to human nature [32-34]. Departmental factors often have commonalities across institutions related to their dependence on the use of resources for enhancing knowledge sharing among workers [35,36]. Evaluating knowledge-sharing behaviors among employees in medical imaging departments at cancer centers depends on identifying the level of awareness of the importance of knowledge sharing and the factors that affect knowledge-sharing practices [37]. These factors are divided into facilitators and barriers. On the basis of a previous study by Almashmoum et al [38], we can divide the facilitators of knowledge sharing in medical imaging departments in cancer centers into 3 categories—individual, departmental, and technological facilitators—and the barriers that affect knowledge sharing into 4 categories—individual, departmental, technological, and geographical. Table 1 shows these factors and their components [38]. On the basis of these factors, we sought to identify awareness of knowledge-sharing behaviors, evaluate the factors affecting knowledge sharing in the medical imaging departments of 2 cancer centers (The Christie and the Kuwait Cancer Control Center [KCCC]), and compare results across these 2 centers.

**Table 1:** Factors that affect knowledge-sharing practices in medical imaging departments [38].

			Factors
**Facilitators**			
	Individual facilitators	Trust Positive attitudes Awareness Experience Intrinsic motivation Personality Self-esteem Self-efficacy	
	Departmental facilitators	Multidisciplinary team and community of oncologists Leadership Culture Teamwork Extrinsic motivation Learning and training Physician rounds Departmental arrangements	
	Technological facilitators	Information and communications technology (picture archiving and communication system, social media, intranet, extranet, telemedicine, and teleradiology) Network Digital library	
**Barriers**			
	Financial barriers	Cost	
	Administrative barriers	Language Time Shortage of staff Lack of transparency Lack of experience	
	Technological barriers	Low-speed network Upgrade system Lack of equipment	
	Geographical barriers	Geographical distance	

### An Overview of The Christie and KCCC

Cancer centers are tertiary care units performing diagnostic scans and therapeutic treatments, blood testing, histology, stem cell laboratory tests, and palliative care related to cancer. The Christie is the largest single cancer center in Europe. It is located in Manchester, United Kingdom; serves 3.2 million people across the Greater Manchester region; and provides treatment for >60,000 patients with cancer per year [39].

The KCCC was established in 1968 and is one of the largest centers in Kuwait that provides comprehensive care for patients with cancer. Its main mission is to focus on improving physician and nursing education. More than 2000 patients with cancer are treated annually at the KCCC [40].

### Objectives

This study had several objectives. The purpose of this study was to evaluate knowledge-sharing practices among health care professionals at The Christie and KCCC, as well as identifying the current knowledge-sharing mechanisms and the facilitators and barriers that affect knowledge sharing among health care professionals in the medical imaging departments of the aforementioned 2 cancer centers. From this, we aimed to construct a new definition of knowledge sharing as it relates to medical imaging departments. Finally, we aimed to draw conclusions on how to improve knowledge-sharing practices and their effects on the quality of patient services.

### Research Questions

There are several challenges in communication among health care professionals, such as lack of awareness of the importance of knowledge-sharing behaviors and factors that affect knowledge-sharing practices. This affects knowledge-sharing behaviors and, therefore, knowledge management implementation, which is considered important in any health care institution to perform tasks for patients. There are several questions raised based on that observation:

1. What is the level of motivation for knowledge sharing among employees in health care institutions?
2. What current knowledge-sharing tools exist in health care institutions?
3. What are the challenges faced by health care professionals related to knowledge sharing?
4. How can knowledge-sharing practices in medical imaging departments be improved?
5. What are the factors that affect knowledge sharing among health care professionals?
6. What is the definition of knowledge sharing in medical imaging departments?

## Methods

### Research Design and Sampling Techniques

This study was performed using a concurrent cross-sectional triangulation mixed methods design, which combined online semistructured interviews with health care professionals who worked in medical imaging departments and an electronic survey that was distributed among health care professionals who worked at The Christie and KCCC to evaluate knowledge-sharing practices and identify factors that affect knowledge-sharing behaviors. This study was conducted between February 2023 and July 2023. The sampling techniques that were used for the selection of health care professionals for the questionnaire and semistructured interviews were self-selection sampling and snowball sampling, respectively. A self-selection approach was applied to select participants who indicated a desire to take part in the research [41]. Self-selection sampling was used for the questionnaires and snowball sampling was used for the semistructured interview because this study evaluated knowledge-sharing practices in medical imaging departments. The questionnaire was distributed among professionals using a WhatsApp group (Meta Platforms) at the KCCC and on the internal page of The Christie. Snowball sampling focused on a group of people who had the same specialties to participate in the interviews. There were several specialties in this department, such as physicians, technologists, senior managers, the head of the department, nurses, and radiotherapists.

### Ethical Considerations

This study followed the University of Manchester’s ethical guidelines. The ethics committee of The Christie determined that the study did not require ethical approval based on the official decision tool of the University of Manchester because the study was conducted with professionals and did not require sensitive questions, vulnerable groups, or risk of disclosures of anonymized information. Whereas the KCCC did require ethical approval especially for them, which it provided (3797). All respondents were health care professionals who signed a consent form to participate in the questionnaires and audio-recorded semistructured interviews. The consent form explained the purpose of the study as well as any other information that the participants might require. In addition, the health care professionals’ personal information was kept anonymous and confidential.

### Quantitative Methods and Data Analysis

The purpose of this questionnaire was to evaluate the level of awareness of the importance of knowledge-sharing practices in medical imaging departments and the factors that affect knowledge-sharing behaviors. The questions were derived from previous related studies on knowledge sharing and modified to fit the overall research aim and answer the research questions [42-44]. The questionnaire items were written in English. They consisted of both nominal and ordinal scales. The entire questionnaire that was used in this study can be found in Multimedia Appendix 1. It was divided into 6 sections, as shown in Textbox 1. It consisted of 66 questions, with an additional open-ended question at the end. A pretest study was conducted among 10 academic lecturers and PhD students at the University of Manchester. The purpose of the pretest study was to make sure that the questionnaire items could be understood in health care institutions. After that, the electronic questionnaires were distributed among health care professionals at the KCCC using WhatsApp and among health care professionals working at The Christie through a posting on the hospital’s intranet. After the collection of the quantitative data, an analysis was performed using the Qualtrics XM software package (Qualtrics International Inc).

**The sections of the questionnaire.**

- Section 1: an overview of knowledge sharing
- Section 2: the consent form (5 statements)
- Section 3: demographic profile of the health care professionals (7 multiple-choice questions)
- Section 4: questions about knowledge-sharing practices and background (4 multiple-choice questions)
- Section 5: questions that examined knowledge-sharing factors (55 questions on a 7-point Likert scale from “strongly disagree” to “strongly agree”)
- Section 6: 1 open-ended question

### Qualitative Methods and Data Analysis

The semistructured interviews were conducted on the web using Microsoft Teams at the same time as the distribution of the questionnaires. The interviews started with brief introductory remarks about knowledge sharing followed by questions that related to the health professionals’ experiences and definitions of knowledge sharing. After that, the questions were designed to assess factors and practices related to knowledge sharing. Multimedia Appendix 1 presents the consent form and the interview questions. The semistructured interviews were conducted with health care professionals who were working in the medical imaging departments of both cancer centers. Each interview took approximately 25 to 45 minutes. Invitations were sent electronically via WhatsApp for KCCC participants and posted on the internal page of The Christie. All the participants signed a consent form related to the interviews and audio recording. Thematic analysis was used to analyze the semistructured interview transcripts. The coding and creation of themes was conducted using the NVivo software (Lumivero).

## Results

### Quantitative Analysis

#### Demographic Characteristics and Response Rate

A total of 77 health care professionals from The Christie participated in this study with a 100% (77/77) response rate based on the statistical analysis using the Qualtrics XM software. In total, 38% (29/77) of the respondents answered all the survey questions. In addition, all of them worked in the medical imaging department. The response rate from the KCCC was 100% (145/145), of whom 48% (70/145) from all departments completed all survey questions. A total of 80% (56/70) of the health care professionals who participated were from the medical imaging department at the KCCC. The demographic characteristics for both centers are shown in Table 2.

**Table 2:** Demographic characteristics.

		KCCC^a^ (n=56), n (%)	The Christie (n=29), n (%)
**Sex**			
	Male	26 (46)	7 (24)
	Female	29 (52)	20 (69)
	Prefer not to say	0 (0)	2 (7)
**Age group (y)**			
	<20	0 (0)	1 (3)
	20-30	3 (5)	8 (28)
	30-40	19 (34)	7 (24)
	40-50	25 (45)	8 (28)
	50-60	7 (12)	3 (10)
	>60	2 (4)	2 (7)
**Educational level**			
	Diploma	7 (12)	4 (14)
	First degree (bachelor’s)	26 (46)	12 (41)
	Master’s degree	11 (20)	8 (28)
	Doctorate	9 (16)	0 (0)
	Other	3 (5)	5 (17)
**Work experience (y)**			
	<10	18 (32)	22 (76)
	10-20	29 (52)	6 (21)
	20-30	7 (12)	1 (3)
	>30	2 (4)	0 (0)

^a^KCCC: Kuwait Cancer Control Center.

#### Knowledge-Sharing Practices

Knowledge sharing is defined as sharing ideas, thoughts, and experiences among health care professionals to create new knowledge. Sharing knowledge requires several facilitators to accelerate knowledge-sharing behaviors, for example, in morning meeting sessions, multidisciplinary team meetings, and conferences. These practices can improve patient outcomes and minimize medical errors. In addition, they can help make such shared knowledge reusable for all health care professionals. The results at The Christie revealed that 59% (17/29) of health care professionals participated in knowledge-sharing activities available in their department on a daily basis and only 14% (4/29) of health care professionals did not participate in any of those activities. At the KCCC, the results showed that 57% (32/56) of health care professionals participated on a daily basis in the knowledge-sharing activities that were available in their department. On the other hand, only 4% (2/56) of health care professionals never participated in those activities.

#### The Mechanisms of Knowledge-Sharing Practices

Sharing knowledge among health care professionals requires different mechanisms. Those mechanisms were classified into either physical or online tools, for example, face-to-face communication, phone calls, social media, emails, and Microsoft Teams. The results showed that 83% (24/29) of the health care professionals at The Christie used email and face-to-face communication to share their knowledge, whereas 86% (48/56) of the health care professionals at the KCCC used face-to-face communication as the main tool to share their knowledge. In total, 59% (17/29) of the health care professionals at The Christie used Microsoft Teams as a tool to share knowledge. At the KCCC, the results showed that 48% (27/56) of the health care professionals also used phone calls and social media to share their knowledge.

#### Level of Motivation

We examined the motivational level by exploring the level of willingness to share knowledge among health care professionals. Most of the health care professionals in both cancer centers had a high motivational level to practice knowledge-sharing behaviors with their peers at the workplace. The findings showed that 38% (21/56) of the health care professionals at the KCCC were highly motivated compared with 48% (14/29) of the health care professionals at The Christie, as shown in Table 3. This percentage indicated that the level of motivation in both centers among health care professionals was high. Regarding incentives and policies, Table 4 shows that 79% (44/56) of the health care professionals who worked at the KCCC indicated that there were incentives to encourage knowledge-sharing practices, whereas half (15/29, 52%) of the health care professionals who worked at The Christie indicated that there were no incentives to encourage knowledge-sharing practices. This comparison showed that the health care professionals at The Christie had a high motivational level but their department did not have incentives and a clear policy to encourage knowledge-sharing practices, which could affect their engagement in these practices.

**Table 3:** Motivational level of the health care professionals at The Christie and Kuwait Cancer Control Center (KCCC).

	Very low, n (%)	Low, n (%)	Medium, n (%)	High, n (%)	Very high, n (%)
The Christie (n=29)	2 (7)	0 (0)	6 (21)	14 (48)	7 (24)
KCCC (n=56)	0 (0)	5 (9)	14 (25)	21 (38)	16 (29)

**Table 4:** Comparison of incentives or polices in place to encourage knowledge sharing between The Christie and the Kuwait Cancer Control Center (KCCC).

	Yes, n (%)	No, n (%)
The Christie (n=29)	15 (52)	14 (48)
KCCC (n=56)	44 (79)	12 (21)

#### Factors Affecting Knowledge-Sharing Practices

The questionnaire evaluated the factors that affect knowledge sharing in medical imaging departments. On the basis of prior work, we identified 19 factors that affect knowledge-sharing behaviors among health care professionals working in medical imaging departments [38]. These factors are divided into 3 categories: individual, departmental, and technological. Individual factors comprise 8 components (trust, positive attitudes, awareness, experience, personality, intrinsic motivation, self-esteem, and self-efficacy). Departmental factors comprise 8 components (community of practice, leadership, culture, teamwork, extrinsic motivation, learning and training, physician rounds, and departmental arrangements). Technological factors comprise 3 components (ICT, network, and digital technology). These factors were measured on a 7-point Likert scale using an equivalent interval of 6/7=0.86. Multimedia Appendix 2 shows the mean score for each component. The mean score was classified as follows: “strongly disagree” for values within the range of 1.00 to 1.86, “disagree” for values within the range of 1.86 to 2.72, “somewhat disagree” for values within the range of 2.72 to 3.58, “neither agree or disagree” for values within the range of 3.58 to 4.44, “somewhat agree” for values within the range of 4.44 to 5.3, “agree” for values within the range of 5.30 to 6.16, and “strongly agree” for values within the range of 6.16 to 7 [28]. All the Likert scale results can be found in Multimedia Appendices 3 and 4 for The Christie and KCCC, respectively.

At the KCCC, the values for all the components of the factors that affect knowledge-sharing behaviors fell between “agree” and “strongly agree.” At The Christie, the values fell between “somewhat agree” and “strongly agree.”

Individual factors are important for enhancing knowledge-sharing practices among health care professionals. Trust plays a vital role in knowledge-sharing practices. It creates a strong relationship among health care professionals. In medical imaging departments, trust between the senior manager and health care professionals and among health care professionals is vital to share knowledge smoothly and provide high-quality health care services. The mean scores for trust were 5.7 and 5.51 in both centers, which corresponds to “agree.” Thus, more than half of the health care professionals had a level of trust that could improve knowledge-sharing behavior by building trust relationships among each other. Awareness of the importance of knowledge sharing helps increase knowledge-sharing behaviors in daily work. In addition, knowledge sharing helps health care professionals gain new knowledge, fosters learning, and prevents repetitive mistakes. The results showed that the mean score for awareness was 6.42 at the KCCC, which corresponds to “strongly agree,” versus 5.48 at The Christie, which corresponds to “agree.” Therefore, health care professionals in both centers had a clear awareness of the importance of knowledge sharing in improving their skills and health services. Health care professionals in both cancer centers believed that positive attitudes help enhance knowledge-sharing behaviors. The results showed that the mean scores for positive attitudes in both cancer centers were 6.4 and 6.16, which correspond to “strongly agree.” Health care professionals having good experience increases knowledge-sharing behaviors. The mean scores for experience were 6.2 at the KCCC and 5.77 at The Christie, which correspond to “strongly agree” and “agree,” respectively. Therefore, health care professionals in both centers believed in the importance of experience in enhancing knowledge sharing. In addition, they had enough experience that could help them share it with others. Health care professionals in both centers had an extroverted personality, which opens to others and allows them to share knowledge with others. As shown in Multimedia Appendix 2, the mean scores for personality were 6.15 for KCCC and 6.18 for The Christie, which correspond to “agree” at the KCCC and “strongly agree” at The Christie. Hence, the results showed that most of them have self-confidence and felt open to new ideas when they practiced knowledge sharing. Self-esteem and self-efficacy are the main components of the individual factors. The mean scores for these components in both cancer centers were close to “agree,” which means that health care professionals have self-efficacy and self-esteem regarding their ability to successfully share knowledge with their peers. Finally, regarding intrinsic motivation, the mean scores were 5.96 at the KCCC and 5.89 at The Christie, which means that most of the health care professionals had intrinsic motivation that allowed them to share their knowledge.

Health care institutions are considered knowledge-based environments due to the large amount of knowledge, either tacit or explicit, that needs to be managed. To maximize the benefit of that knowledge, each department in a health care institution has a responsibility to share knowledge among their professionals. Extrinsic motivation is important to enhance knowledge-sharing practices through providing acknowledgment, appreciation, incentives, and bonuses to health care professionals. The results showed that the mean score for extrinsic motivation was 5.66, which corresponds to “agree,” at the KCCC. Thus, health care professionals who worked at the KCCC received motivation from their senior managers. In contrast, the mean score was 4.37 at The Christie, which corresponds to “neither agree nor disagree.” This implies an absence of departmental encouragement of knowledge-sharing behaviors, as the respondents believed in the importance of extrinsic motivation in enhancing knowledge sharing. Health care professionals in both cancer centers believed that the leadership plays a significant role in providing encouragement, improving knowledge-sharing activities, and minimizing conflict. The mean scores for leadership in both cancer centers were close to “agree,” which means that the leadership in both cancer centers had a positive impact in enhancing knowledge sharing. Working and learning as a team is important to enhance knowledge-sharing practices. Health care professionals in both centers believed in the importance of teamwork for increasing knowledge-sharing practices. The mean scores for teamwork in both cancer centers were close to “strongly agree,” which means that they worked as a team to increase productivity. Both cancer centers had a culture of communication to enhance knowledge sharing, with mean scores close to “strongly agree.” The community of practice consists of several communities that specialize in making decisions in specific cancer centers. In addition, multidisciplinary team meetings are one of the most prominent types of meetings in cancer centers. The mean scores for community of practice in both cancer centers corresponded to “agree,” which means that, in both centers, there are several specialized meetings that play important roles in making decisions by enhancing knowledge-sharing behaviors. Learning and training activities, such as workshops, lectures, and conferences, play a vital role in enhancing knowledge sharing. At the KCCC, the mean score for this component was 5.79, which corresponds to “agree.” Thus, health care professionals participated widely in several learning and training activities to enhance their skills. In contrast, at The Christie, the mean score for this component was 5.25, which corresponds to “somewhat agree.” Thus, health care professionals received limited learning and training support to increase their skills. At the KCCC, there were enough empty rooms and open space to enhance knowledge sharing, with a mean score of 6.1 for departmental arrangements, which corresponds to “agree,” compared with not enough space at The Christie, with a mean score of 5.29 for departmental arrangements, which corresponds to “somewhat agree.” Daily physician rounds play a vital role in developing health care professionals’ skills. The mean score for this component was 5.64 at the KCCC, which corresponds to “agree,” and 4.82 at The Christie, which corresponds to “somewhat agree.” Therefore, health care professionals at the KCCC believed in the importance of physician rounds in enhancing knowledge sharing more than professionals at The Christie.

Technological factors are considered a dynamo of knowledge-sharing practices. High-speed networks play a significant role in enhancing knowledge-sharing practices. Both cancer centers had a high-speed network and believed in its importance, with mean scores of 5.33 at the KCCC and 5.56 at The Christie, which correspond to “agree.” ICT is crucial to support sharing information. Intranet, extranet, PACS, and social media are considered types of ICT. In addition, ICT requires skills to use it and maintenance to address and report any related problems. At the KCCC, the mean score for ICT was 5.62, which corresponds to “agree.” Thus, in the medical imaging departments, there were enough ICT infrastructures that they used to share their knowledge, and health care professionals were trained well to use those technologies and report any problems they faced. At The Christie, the mean score for ICT was 5.20, which corresponds to “somewhat agree.” Thus, in the medical imaging departments, health care professionals used ICT to share their knowledge, and they had enough skills to use it. However, they did not frequently use social media to share their knowledge compared to health care professionals at the KCCC. To access to updated articles and resources, digital libraries were a valuable tool that allowed health care professionals to gain new information to share their knowledge with each other. The mean scores for this component were 5.91 at the KCCC and 5.62 at The Christie, which correspond to “agree.” Thus, health care professionals in both cancer centers believed in the importance of digital libraries in enhancing knowledge sharing.

### Qualitative Analysis

#### Overview of Analysis

Semistructured interviews were used to gather the background experience of health care professionals who worked in the medical imaging department (heads of department, technologists, nurses, physicians, and radiotherapists). The data were used to validate the quantitative data. A total of 13 health care professionals participated in this part of the study. Of the 13 participants, 11 (85%) were from the KCCC, and only 2 (15%) were from The Christie. The outcomes of the online semistructured interviews shed light on 3 themes that related to knowledge-sharing practices, such as definitions of knowledge sharing, factors, and challenges to knowledge-sharing behaviors.

#### Theme 1: Definition of Knowledge Sharing in Medical Imaging Departments

Despite the fact that the term *knowledge sharing* is not new, most of the health care professionals asked for clarification of the term before giving their definition based on their experience in the department. In general, most of them gave the proper definition of knowledge sharing in the medical imaging department; Multimedia Appendix 5 shows their definitions. On the basis of their views, the general key points to structure the definition of knowledge sharing are as follows:

1. All health care professionals have a specific amount of knowledge derived from their studies and experience.
2. Knowledge sharing involves sharing information and updated protocols and circulars among health care professionals.
3. Knowledge sharing takes places between colleagues or among a wider team in one department or with professionals from another hospital.
4. Knowledge sharing occurs through various activities, such as lectures, workshops, meetings, and conferences.
5. There is no benefit from keeping knowledge to oneself. It remains inactive until it is shared.
6. Knowledge sharing among health care professionals helps patients obtain a more accurate diagnosis.

#### Theme 2: Factors Affecting Knowledge Sharing

The findings of a deep analysis of the qualitative data were consistently validated by the quantitative data. The factors that affect knowledge sharing could be classified as facilitators that enhance knowledge sharing and as barriers when those factors have a negative impact on knowledge-sharing behavior. Facilitators are classified into 3 categories: individual factors, departmental factors, and technological factors.

##### Subtheme 2.1: Individual Factors

Participants stated that they were aware of the importance of knowledge-sharing practices in maximizing health services in various specialties. In addition, they were aware of the importance of sharing knowledge with other peers to apply it in various situations. Participant C indicated the following:

Knowledge sharing is very important amongst clinicians of various specialities and various field.

Most health care professionals had a variety of skills and experience that they had gained throughout their careers. Their skills and experience allowed them to share their knowledge with other peers who had less experience. However, some of them felt shy about sharing their knowledge with others in a large group. In addition, sharing knowledge among health care professionals depends on the personality of those doing the sharing. Participant B said the following:

And lots of knowledge, but those kinds of people also they do not want to share it in public among larger group of audience or larger group of attendees. Things can be accomplished by dealing with getting the information from a group in a way that they do not feel uncomfortable in sharing their ideas.

##### Subtheme 2.2: Departmental Factors

According to the findings of this data analysis, there are several factors related to departmental factors. Knowledge-sharing practices increased with the ability of the department to foster a culture of communication that allowed health care professionals to share their knowledge. Participant C indicated the importance of creating a culture to support sharing knowledge:

So definitely there is a good positive environment of knowledge sharing and knowledge building also...sharing information maybe I think it changes the culture of the team if we can inspire people to learn and share more.

In addition, working as a team helps enhance knowledge-sharing practices because health care professionals work in groups to perform specific tasks and procedures. The leadership plays an important role in enhancing knowledge sharing by establishing clear policies for sharing knowledge and allowing health care professionals to participate in several activities. Those activities fall under the component of learning and training. Most respondents indicated that there were several activities available, such as presenting and attending lectures, attending conferences locally and internationally, participating in training sessions and workshops, creating posters, attending seminars, and engaging in continuing education. In addition, respondent C suggested participating in journaling sessions to keep up to date on information and maximize their knowledge, allowing knowledge-sharing with others to improve treatment plans. Extrinsic motivation is divided into 2 categories: physical and emotional incentives. In this regard, participants B and F indicated that excellent evaluations and other signs of appreciation, such as receiving certificates or having their names added to papers, posters, or lectures prepared by professionals, were an effective means of encouragement. A community of practice involves specialized meetings among health care professionals, including multidisciplinary team meetings. These high-level knowledge-sharing meetings can involve decisions on treatment plans for patients with cancer. All health care professionals in different disciplines play a certain role in interpreting the final treatment plan. Therefore, attending these meetings helps them develop their skills by gaining new knowledge. However, a few of the respondents from KCCC indicated that they did not have any idea about those meetings and they were not involved in them at all.

##### Subtheme 2.3: Technological Factors

Technological factors are considered a dynamo of knowledge-sharing practices. The results revealed that there were several types of ICT infrastructure in both departments, such as PACS, the bleep system, hospital information system, registration information system, and social media apps, in addition to online communication tools such as Zoom and Microsoft Teams. The use of online tools became prevalent after the COVID-19 pandemic to maintain communication among health care professionals by setting online meetings, videoconferences, and online circular discussions for better patient services and to protect their lives. As a consequence, most of the respondents preferred online tools over face-to-face communication for several reasons, such as availability, time saving, and removing geographical barriers. On the other hand, most of the participants indicated that using online tools requires positive attitudes toward technology, maintenance, and a high-speed network to keep the tools active for sharing knowledge. Participants C and K indicated the following:

I would say sometimes because of some Internet issues or some Internet connection, yes. So it becomes difficult. Sometimes it takes time for the reports to be automatically uploaded into HIS.

#### Theme 3: The Challenges to Knowledge-Sharing Behaviors

The in-depth analysis of the participants’ views brought about some of the challenges that health care professionals face in practicing knowledge sharing. They mentioned that a lack of staff is one of the challenges that managers face, which results in a lot of duties in daily work, influencing knowledge sharing. This prevents them from practicing knowledge-sharing activities. Participant B indicated the following:

I think this is actually one of the problem in the cancer Control Center is that you don’t have we don’t have enough stuff for the number of the service that we are doing so it it’s you know one can imagine that three or five physician do a delivery of probably 50 or 60 clinical service a day for the whole 365 days a year and we are expected to give our time to knowledge share as reserving one or two hours per week knowledge sharing when we are expected to finalize the clinical service, this is the problem.

In addition, time constraints were a main challenge to knowledge sharing that participant C mentioned:

We wish we had more time for knowledge sharing because definitely it is very useful, but maybe we’re not able to do it as often or as for a longer period of time because of the time constraint. Yeah. Our challenge I would say one is the time that we would want, definitely we don’t have. I feel that we don’t have enough time to sort of have more of discussing of cases. Interacting with more with each other because many times when I have faced that that I want an opinion but there’s just no time for me or for the other person to actually look at the case and go into details and try to get some information and then. So, we tend to sort of maybe cut down on the sessions and use that time to report our cases. So, I feel that we should have more dedicated time. can make sure that the message delivered to him that he will ask his colleague or there is a minute. Of course, we can share it with him.

In addition to these challenges, there were challenges related to a person’s attitudes. For instance, some were less interested in sharing their knowledge because of a lack of awareness of the importance of knowledge sharing. Some lacked awareness regarding who was responsible for sharing knowledge and were not aware of the benefits of sharing their knowledge. In addition, according to participant L, “It’s a cooperation. I said yes, this is one challenge and then also communication with the patient.”

## Discussion

### Principal Findings

Health care institutions are knowledge-based environments where knowledge-sharing practices are an important step to achieve good knowledge management. This can help these departments reach their intended goals, mission, vision, and objectives for the delivery of high-quality health services. The objectives of this study were to evaluate the factors that affect knowledge sharing in the medical imaging departments of 2 cancer centers, identify the current tools for knowledge-sharing practices, structure a new definition of knowledge sharing, assess the challenges, and identify the areas for improvement in knowledge-sharing behaviors. On the basis of the respondents’ views and thoughts on knowledge sharing during the semistructured interviews, we can define knowledge sharing as follows: “All health care professionals have a certain amount of knowledge built up through their work in a specific field. That knowledge (either tacit or explicit) will remain inactive until they share it with their peers or the wider team through meetings, lectures, and workshops and confirm that knowledge, or recreate new knowledge to better help patients.”

The results showed that the level of motivation of the health care professionals to share their knowledge in their daily work was high in both cancer centers. There are several studies that suggest the same results regarding the high level of motivation toward knowledge sharing [20,37]. There were several mechanisms for sharing knowledge among health care professionals. The findings illustrated that the current mechanisms of knowledge sharing at The Christie were face-to-face communication and email, each with an equal percentage of participants (24/29, 83%), compared with the use of face-to-face communication as a main tool to share knowledge at the KCCC (48/56, 86%). This agrees with research showing that radiologists prefer face-to-face communication to share their thoughts and interests [45]. In addition, using social media as a tool for sharing knowledge among health care professionals was a common practice at the KCCC, for instance, using WhatsApp as a main tool for internal circulation of information, announcements, and updates regarding protocol. Most respondents expressed that using social media as a online tool is faster than using traditional tools. In terms of availability, the information will remain in an app that can be accessed at any time because most health care professionals are busy with cases and do not have enough time to attend meetings face-to-face. Informing other people about key points of the meetings using those apps helps enhance knowledge-sharing practices among health care professionals. However, the use of social media apps in health care institutions still appears to be more prevalent in the West compared to the East [37,46-48]. During the COVID-19 pandemic, the use of online tools for sharing knowledge came to play a vital role in keeping knowledge circulating among health care professionals and making communication with others safe [49]. There is evidence to suggest that these practices led to an increase in positive patient outcomes and health services during the crisis [49,50]. Using hybrid tools contributes to the ability to share knowledge in a way that suits health care professionals [50].

The health care professionals in this study responded “strongly agree” or “somewhat agree” to questions on the importance of the examined knowledge-sharing practices. Therefore, this study found that health care professionals in both cancer centers believed in the importance of factors that affect knowledge sharing in enhancing knowledge-sharing practices in their daily work. Several studies support this finding [20-31]. However, the mean score for extrinsic motivation at The Christie was 4.37, which corresponds to “neither agree or disagree,” suggesting that the department may not have given enough encouragement for knowledge-sharing practices. This is reflected in the answers to the questions on encouragement and incentives at The Christie. More than half (15/29, 52%) of the respondents at The Christie indicated that there were no incentives to encourage knowledge-sharing practices. Therefore, health care professionals might intend to avoid participating in knowledge-sharing practices, which has a negative impact on practicing knowledge sharing in general.

In addition to those factors, there were several challenges that affected knowledge-sharing practices addressed by respondents in the semistructured interviews at both cancer centers, such as time constraints and attitudes toward knowledge sharing. In addition, at the KCCC, the respondents addressed language as the main challenge in sharing knowledge because the environment consists of international workers, which means that they communicate with their colleagues using their second language. Previous studies have shown that language is the main barrier that limits knowledge-sharing practices [33,34,37,51]. Fatahi et al [52] have illustrated that language is the first route for communication. Therefore, using a universal language that allows all health care professionals to communicate with their peers is important to enhance knowledge-sharing practices.

To improve knowledge-sharing practices, senior managers suggested in the semistructured interviews that creating a clear policy to share knowledge in the department is important in enhancing knowledge-sharing practices, starting with increasing awareness of the importance of knowledge sharing, followed by encouraging participation in various learning and training activities; improving attitudes to using technology; and, finally, providing encouragement via physical incentives (eg, bonuses and promotions) and emotional incentives (eg, excellent evaluations, certificates of appreciation, or adding an individual’s name to a research paper or lecture). Moreover, hiring more staff helps enhance knowledge sharing by reducing the workload and giving staff members time to share their knowledge and participate in several activities.

### Limitations

There were several limitations to this study that need to be addressed. The sample from The Christie was small in both parts of the study compared to the sample from the KCCC. There was a lack of staff, which limited their participation in the questionnaires and interviews due to work pressures. This study focused only on medical imaging departments and was limited to only those who worked in those departments. Therefore, this study could not evaluate the level of motivation and factors that affect knowledge sharing in all departments in both centers. Future work needs to assess the level of knowledge-sharing practices. A better approach may be to create a maturity model for knowledge sharing to assess the level of maturity and help managers put a clear plan and policy in place to manage knowledge-sharing practices.

### Conclusions

This concurrent mixed methods study provides an evaluation of the factors that affect knowledge sharing in medical imaging departments. In addition, it structured a definition of knowledge sharing in medical imaging departments. On the basis of the questionnaires, health care professionals used face-to-face communication as a main mechanism to share knowledge within these departments at The Christie and KCCC (24/29, 83% and 48/56, 86%, respectively). Therefore, using knowledge-sharing mechanisms within departments for the purpose of enhancing knowledge-sharing practices is vital to speed up the knowledge-sharing process. In addition, health care professionals in the medical imaging departments in both centers had a good personality, positive attitudes, trust, a high self-esteem, and self-efficacy. Therefore, the intention to share knowledge was high in both cancer centers based on the individual factors. In the medical imaging departments, there were health care professionals that led the process of knowledge sharing by creating a culture of communication, setting several meetings, and giving everyone an equal opportunity to participate in learning and training activities. However, regarding extrinsic motivation, half (15/29, 52%) of the respondents at The Christie indicated that there was a lack of incentives, which was reflected in the low mean score for this component. Therefore, establishing a clear plan and providing incentives has a positive impact on knowledge-sharing practices. ICT infrastructures were available in both cancer centers, with a high-speed network to run those technologies. Most of the respondents in the semistructured interviews addressed several challenges of knowledge sharing, such as language barriers and a lack of time, staff, and a clear plan for knowledge sharing. Finally, this study provides managers with an evaluation of the factors that affect knowledge sharing in both cancer centers and allows them to address the challenges and improve them.

## Appendix

Multimedia Appendix 1 The questionnaires items, consent form for the interview, and the questions of the interviews.

Multimedia Appendix 2 Results for the factors that affect knowledge sharing in both cancer centers.

Multimedia Appendix 3 Evaluation of factors at The Christie.

Multimedia Appendix 4 Evaluation of factors at the Kuwait Cancer Control Center.

Multimedia Appendix 5 Definition of knowledge sharing in medical imaging departments based on the respondents’ views.

## Abbreviations

ICT: information and communications technology
KCCC: Kuwait Cancer Control Center
PACS: picture archiving and communication system
