# Supplementary material for: Evaluating Factors Affecting Knowledge Sharing Among Health Care Professionals in the Medical Imaging Departments of 2 Cancer Centers: Concurrent Mixed Methods Study
Source: JMIR Hum Factors. 2024 Nov 13;11:e53780. doi: 10.2196/53780 (PMC11602759; doi:10.2196/53780)
Supplement: Multimedia Appendix 3 [file humanfactors_v11i1e53780_app3.docx]

**Multimedia Appendix 3. Results for the factors that affect knowledge sharing in The Christie**

**For the following questions please tick (√)one answer only which indicates your extent of agreement: This section examines facilitators of Knowledge sharing. These facilitators are classified into three categories: individuals, departmental, and technological facilitators.**

| Knowledge sharing facilitators | | | | | | | | |  |
| --- | --- | --- | --- | --- | --- | --- | --- | --- | --- |
| Individuals Facilitators | | **Strongly disagree**  **(1)** | **Disagree**  **(2)** | **Somewhat disagree**  **(3)** | **Neither agree or disagree**  **(4)** | **Somewhat agree**  **(5)** | **Agree**  **(6)** | **Strongly agree**  **(7)** | **Mean scare** |
| 10. | **In the hospital, there are periodic meetings in which employees**  **working in different disciplines, may participate.** | **2**  **2** | **1**  **2** | **1**  **3** | **3**  **12** | **5**  **25** | **13**  **78** | **4**  **28** | **150**  **(5.17)** |
| 11. | **In the hospital, there are continuous education programmes such as training courses and workshops within hospital, in which employees can participate.** | **0**  **(0)** | **1**  **(2)** | **1**  **(3)** | **4**  **(16)** | **3**  **(15)** | **6**  **(24)** | **14**  **(98)** | **158**  **(5.44)** |
| Total | | **2**  **(2)** | **2**  **(4)** | **2**  **(6)** | **7**  **(28)** | **8**  **(40)** | **19**  **(114)** | **18**  **(126)** | **320**  **(5.51)** |
|  | **Trust** |  |  |  |  |  |  |  |  |
| 12. | **I feel fully confident in my own knowledge and I want to share it with others.** | **0**  **(0)** | **0**  **(0)** | **1**  **(3)** | **1**  **(4)** | **12**  **(60)** | **9**  **(54)** | **6**  **(42)** | **163**  **(5.6)** |
| 13. | **I trust in knowledge of my colleagues and information that they shared with me.** | **0**  **(0)** | **0**  **(0)** | **2**  **(6)** | **1**  **(4)** | **6**  **(30)** | **14**  **(84)** | **6**  **(42)** | **166**  **(5.72)** |
| 14. | **If I share my knowledge, my colleagues will feel confident about my ideas, skills, and capabilities to enhance knowledge sharing.** | **0**  **(0)** | **0**  **(0)** | **1**  **(3)** | **3**  **(12)** | **7**  **(35)** | **13**  **(78)** | **5**  **(35)** | **163**  **(5.6)** |
| 15. | **If I faced problems during work, my colleagues will try to help me resolve them.** | **1**  **(1)** | **0**  **(0)** | **3**  **(3)** | **1**  **(4)** | **6**  **(30)** | **11**  **(66)** | **7**  **(49)** | **153**  **(5.2)** |
| Total | | **3**  **(3)** | **0**  **(0)** | **7**  **(21)** | **6**  **(24)** | **31**  **(155)** | **47**  **(282)** | **24**  **(189)** | **674**  **(5.81)** |
|  | **Awareness** |  |  |  |  |  |  |  |  |
| 17. | **I am aware of the importance of knowledge sharing among employees at workplace** | **0**  **(0)** | **0**  **(0)** | **0**  **(0)** | **1**  **(4)** | **0**  **(0)** | **14**  **(84)** | **14**  **(89)** | **177**  **(6.1)** |
| 18. | **Knowledge sharing among employees helps to prevent mistakes that could happen during day-to-day work.** | **1**  **(1)** | **0**  **(0)** | **1**  **(3)** | **1**  **(4)** | **1**  **(5)** | **9**  **(54)** | **16**  **(112)** | **197**  **(6.172)** |
| 19. | **Sharing knowledge with my colleagues will help me to gain new skills, ideas.** | **1**  **(1)** | **0**  **(0)** | **0**  **(0)** | **1**  **(4)** | **3**  **(15)** | **11**  **(66)** | **13**  **(91)** | **177**  **(6.1)** |
| 20. | **Knowledge sharing behaviours help employees to learn faster** | **1**  **(1)** | **1**  **(2)** | **0**  **(0)** | **0**  **(0)** | **0**  **(0)** | **14**  **(84)** | **13**  **(91)** | **178**  **(6.13)** |
| Total | | **3**  **(3)** | **1**  **(2)** | **1**  **(3)** | **3**  **(12)** | **4**  **(20)** | **34**  **(204)** | **56**  **(392)** | **636**  **(5.48)** |
|  | **Positive attitudes** |  |  |  |  |  |  |  |  |
| 21. | **I believe that positive attitudes will help to enhance knowledge sharing among others** | **0**  **(0)** | **1**  **(2)** | **0**  **(3)** | **0**  **(4)** | **2**  **(10)** | **12**  **(72)** | **14**  **(98)** | **189**  **(6.517)** |
| 22. | **Positive attitudes have a significant role in increasing knowledge sharing behaviours** | **0**  **(0)** | **1**  **(2)** | **0**  **(0)** | **1**  **(4)** | **2**  **(10)** | **11**  **(66)** | **14**  **(98)** | **178**  **(6.13)** |
| 23. | **I believe that positive attitudes are the first step to sharing knowledge** | **0**  **(0)** | **1**  **(2)** | **1**  **(3)** | **1**  **(4)** | **3**  **(5)** | **11**  **(66)** | **12**  **(84)** | **164**  **(5.65)** |
| Total | | **0**  **(0)** | **3**  **(6)** | **1**  **(3)** | **2**  **(8)** | **7**  **(35)** | **34**  **(204)** | **40**  **(280)** | **536**  **(6.16)** |
|  | **Experience** |  |  |  |  |  |  |  |  |
| 24. | **I have a good amount of experience that I can share it with my colleagues.** | **0**  **(0)** | **0**  **(0)** | **0**  **(0)** | **1**  **(4)** | **5**  **(25)** | **16**  **(112)** | **7**  **(49)** | **190**  **(6.55)** |
| 25. | **I believe that experience plays a significant role in sharing knowledge** | **0**  **(0)** | **1**  **(2)** | **1**  **(3)** | **0**  **(0)** | **4**  **(20)** | **15**  **(90)** | **8**  **(56)** | **171**  **(5.89)** |
| 26. | **Knowledge sharing behaviours will increase when employees have enough experience.** | **2**  **(2)** | **1**  **(2)** | **1**  **(3)** | **1**  **(4)** | **2**  **(10)** | **18**  **(108)** | **4**  **(28)** | **157**  **(5.4)** |
| Total | | **2**  **(2)** | **2**  **(4)** | **2**  **(6)** | **2**  **(8)** | **11**  **(55)** | **49**  **(294)** | **19**  **(133)** | **502**  **(5.77)** |
|  | **Personality** |  |  |  |  |  |  |  |  |
| 27. | **I have confidence in my ability, to share knowledge.** | **0**  **(0)** | **0**  **(0)** | **0**  **(0)** | **1**  **(4)** | **5**  **(25)** | **15**  **(90)** | **8**  **(56)** | **175**  **(6.03)** |
| 28. | **I enjoy sharing my knowledge with colleagues.** | **0**  **(0)** | **0**  **(0)** | **0**  **(0)** | **1**  **(4)** | **3**  **(15)** | **15**  **(90)** | **10**  **(70)** | **179**  **(6.17)** |
| 29. | **I am open minded and receptive to new ideas.** | **0**  **(0)** | **0**  **(0)** | **0**  **(0)** | **0**  **(0)** | **3**  **(15)** | **13**  **(78)** | **13**  **(91)** | **184**  **(6.34)** |
| Total | | **0**  **(0)** | **0**  **(0)** | **0**  **(0)** | **2**  **(8)** | **11**  **(55)** | **43**  **(258)** | **31**  **(217)** | **538**  **(6.18)** |
|  | **Self-esteem** |  |  |  |  |  |  |  |  |
| 30. | **I believe that self-esteem is an important aspect in sharing knowledge** | **0**  **(0)** | **0**  **(0)** | **0**  **(0)** | **4**  **(16)** | **3**  **(15)** | **12**  **(72)** | **10**  **(70)** | **173**  **(5.965)** |
| 31. | **I have confidence in my ability to successfully share knowledge with colleagues.** | **0**  **(0)** | **0**  **(0)** | **0**  **(0)** | **2**  **(14)** | **5**  **(25)** | **18**  **(108)** | **4**  **(28)** | **175**  **(6.03)** |
| Total | | **0**  **(0)** | **0**  **(0)** | **0**  **(0)** | **6**  **(24)** | **8**  **(40)** | **19**  **(114)** | **14**  **(98)** | **276**  **(4.75)** |
|  | **Self-efficacy** |  |  |  |  |  |  |  |  |
| 32. | **I believe that self-efficacy is important to motivate us to share knowledge** | **0**  **(0)** | **0**  **(0)** | **0**  **(0)** | **1**  **(4)** | **8**  **(40)** | **17**  **(102)** | **3**  **(21)** | **167**  **(5.75)** |
| 33. | **I have the self-efficacy to share my knowledge with others** | **0**  **(0)** | **0**  **(0)** | **1**  **(3)** | **0**  **(0)** | **9**  **(45)** | **16**  **(96)** | **3**  **(21)** | **165**  **(5.689)** |
| Total | | **0**  **(0)** | **0**  **(0)** | **1**  **(3)** | **1**  **(4)** | **17**  **(85)** | **33**  **(198)** | **6**  **(42)** | **332**  **(5.92)** |
|  | **Intrinsic- motivation** |  |  |  |  |  |  |  |  |
| 34. | **I believe that I have knowledge that will help in increasing productivity.** | **0**  **(0)** | **0**  **(0)** | **1**  **(3)** | **2**  **(8)** | **7**  **(35)** | **14**  **(98)** | **5**  **(35)** | **179**  **(6.172)** |
| 35. | **I feel happy when I am helping my colleagues by sharing my knowledge with them** | **0**  **(0)** | **0**  **(0)** | **0**  **(0)** | **2**  **(8)** | **1**  **(5)** | **18**  **(108)** | **8**  **(56)** | **177**  **(6.10)** |
| Total | | **0**  **(0)** | **0**  **(0)** | **1**  **(3)** | **4**  **(16)** | **8**  **(40)** | **32**  **(192)** | **13**  **(91)** | **342**  **(5.89)** |
| Departmental facilitators | | | | | | |  |  |  |
|  | **Extrinsic motivation** |  |  |  |  |  |  |  |  |
| 36. | **There is acknowledgement for employees who share their knowledge from the hospital** | **5**  **(5)** | **2**  **(4)** | **3**  **(9)** | **6**  **(24)** | **5**  **(25)** | **5**  **(30)** | **3**  **(21)** | **118**  **(4.06)** |
| 37. | **Sharing knowledge will help me to advance in my career.** | **4**  **(4)** | **1**  **(2)** | **0**  **(0)** | **4**  **(16)** | **9**  **(45)** | **8**  **(48)** | **3**  **(21)** | **136**  **(4.68)** |
| Total | | **9**  **(9)** | **3**  **(6)** | **3**  **(9)** | **10**  **(40)** | **14**  **(70)** | **13**  **(78)** | **6**  **(42)** | **254**  **(4.37)** |
|  | **Leadership: leadership is an employee who have responsibilities to manage work among several employees in one department such as: head of department, and senior management.** | | | | | | | |  |
| 38. | **I believe that the hospital leadership has a responsibility to encourage and improve knowledge sharing activity.** | **0**  **(0)** | **0**  **(0)** | **0**  **(0)** | **0**  **(0)** | **0**  **(0)** | **15**  **(90)** | **14**  **(98)** | **188**  **(6.48)** |
| 39. | **I believe that leaderships plays an important role in minimizing conflict** | **0**  **(0)** | **0**  **(0)** | **0**  **(0)** | **0**  **(0)** | **4**  **(20)** | **10**  **(60)** | **15**  **(105)** | **185**  **(6.37)** |
| 40. | **The head of department or senior managementhas a positive impact on enhancing knowledge sharing.** | **0**  **(0)** | **2**  **(4)** | **2**  **(6)** | **1**  **(4)** | **3**  **(15)** | **9**  **(54)** | **12**  **(84)** | **167**  **(5.75)** |
| Total | | **0**  **(0)** | **2**  **(4)** | **2**  **(6)** | **1**  **(4)** | **7**  **(35)** | **34**  **(204)** | **41**  **(287)** | **540**  **(6.20)** |
|  | **Team work** |  |  |  |  |  |  |  |  |
| 41. | **I believe that teamwork has a significant role in sharing knowledge** | **0**  **(0)** | **0**  **(0)** | **0**  **(0)** | **0**  **(0)** | **2**  **(10)** | **11**  **(66)** | **16**  **(112)** | **188**  **(6.48)** |
| 42. | **Teamwork is a part of daily work in each department that enhance knowledge sharing.** | **0**  **(0)** | **2**  **(4)** | **0**  **(0)** | **0**  **(0)** | **0**  **(0)** | **12**  **(72)** | **15**  **(105)** | **181**  **(6.24)** |
| 43. | **Teamwork has a positive impact on enhancing well-being among employees.** | **0**  **(0)** | **1**  **(2)** | **0**  **(0)** | **0**  **(0)** | **0**  **(0)** | **11**  **(66)** | **17**  **(119)** | **187**  **(6.44)** |
| Total | | **0**  **(0)** | **3**  **(6)** | **0**  **(0)** | **0**  **(0)** | **2**  **(10)** | **34**  **(204)** | **48**  **(336)** | **556**  **(6.39)** |
|  | **Culture** |  |  |  |  |  |  |  |  |
| 44. | **I believe that a culture of communicating is important to enhance knowledge sharing.** | **0**  **(0)** | **0**  **(0)** | **0**  **(0)** | **0**  **(0)** | **2**  **(10)** | **13**  **(78)** | **14**  **(98)** | **186**  **(6.41)** |
| 45. | **Cultural collaboration has a significant role in sharing knowledge among employees.** | **0**  **(0)** | **0**  **(0)** | **0**  **(0)** | **1**  **(4)** | **5**  **(25)** | **11**  **(66)** | **12**  **(84)** | **179**  **(6.17)** |
| Total | | **0**  **(0)** | **0**  **(0)** | **0**  **(0)** | **1**  **(4)** | **7**  **(35)** | **24**  **(144)** | **26**  **(182)** | **365**  **(6.29)** |
| Communities of Practice are defined as types of meeting or working groups that takes place among members of a healthcare community from within different fields in order to share knowledge. | | | | | | | | |  |
| 45. | **There are communities of practice in the hospital that I can you for knowledge sharing.** | **1**  **(1)** | **1**  **(2)** | **1**  **(3)** | **8**  **(32)** | **6**  **(30)** | **9**  **(54)** | **3**  **(21)** | **143**  **(4.93)** |
| 46. | **I believe that communities of practice play a significant role in enhancing knowledge sharing among employees** | **0**  **(0)** | **0**  **(0)** | **2**  **(6)** | **6**  **(24)** | **4**  **(20)** | **14**  **(84)** | **3**  **(21)** | **155**  **(4.34)** |
| 47. | **Multidisciplinary team meetings are important to increase patients outcomes and reduce errors.** | **0**  **(0)** | **0**  **(0)** | **0**  **(0)** | **3**  **(12)** | **4**  **(20)** | **9**  **(54)** | **13**  **(91)** | **177**  **(6.10)** |
| Total | | **0**  **(0)** | **0**  **(0)** | **3**  **(9)** | **17**  **(68)** | **14**  **(70)** | **32**  **(192)** | **19**  **(133)** | **472**  **(5.42)** |
|  | **Learning and training** |  |  |  |  |  |  |  |  |
| 48. | **I believe that workshops have a significant impact on knowledge sharing** | **0**  **(0)** | **0**  **(0)** | **0**  **(0)** | **4**  **(16)** | **6**  **(30)** | **15**  **(90)** | **4**  **(28)** | **164**  **(5.655)** |
| 49. | **In the hospital, there are workshops and training sessions that enhance my learning and knowledge sharing.** | **1**  **(1)** | **1**  **(2)** | **2**  **(6)** | **3**  **(12)** | **9**  **(45)** | **7**  **(42)** | **6**  **(42)** | **150**  **(5.17)** |
| 50. | **The hospital encourages employees to participate in conferences locally and internationally.** | **2**  **(2)** | **3**  **(6)** | **3**  **(9)** | **3**  **(12)** | **8**  **(40)** | **5**  **(30)** | **5**  **(35)** | **134**  **(4.62)** |
| 51. | **I believe that morning meeting sessions have a positive impact on knowledge sharing.** | **2**  **(2)** | **0**  **(0)** | **0**  **(0)** | **1**  **(4)** | **7**  **(35)** | **12**  **(72)** | **7**  **(49)** | **162**  **(5.58)** |
| Total | | **5**  **(5)** | **4**  **(8)** | **5**  **(15)** | **11**  **(44)** | **30**  **(150)** | **39**  **(234)** | **22**  **(154)** | **610**  **(5.25)** |
|  | **Departmental arrangements** |  |  |  |  |  |  |  |  |
| 52. | **In the hospital, there is a conference room or meeting room can be used for knowledge sharing.** | **1**  **(1)** | **1**  **(2)** | **3**  **(9)** | **1**  **(4)** | **5**  **(25)** | **8**  **(48)** | **10**  **(70)** | **159**  **(5.48)** |
| 53. | **I believe that offering an open space to share knowledge is part of a department’s responsibility.** | **2**  **(2)** | **0**  **(0)** | **2**  **(6)** | **3**  **(12)** | **3**  **(15)** | **12**  **(72)** | **7**  **(49)** | **156**  **(5.379** |
| 54. | **In the hospital, knowledge sharing practices are part of daily working practice.** | **1**  **(1)** | **1**  **(2)** | **4**  **(12)** | **3**  **(12)** | **7**  **(35)** | **7**  **(42)** | **6**  **(42)** | **146**  **(5.03)** |
| Total | | **4**  **(4)** | **2**  **(4)** | **9**  **(27)** | **7**  **(28)** | **15**  **(75)** | **27**  **(162)** | **23**  **(161)** | **461**  **(5.29)** |
|  | **Doctor rounds** |  |  |  |  |  |  |  |  |
| 55. | **I believe that daily doctor rounds are an important way of improving knowledge.** | **0**  **(0)** | **0**  **(0)** | **0**  **(0)** | **11**  **(44)** | **3**  **(15)** | **10**  **(60)** | **5**  **(35)** | **154/29**  **(5.31)** |
| 56. | **In the hospital, there are daily rounds for professional employees to help develop skills.** | **0**  **(0)** | **0**  **(0)** | **3**  **(9)** | **16**  **(64)** | **0**  **(0)** | **7**  **(42)** | **3**  **(21)** | **136**  **(4.68)** |
| Total | | **0**  **(0)** | **0**  **(0)** | **3**  **(9)** | **27**  **(108)** | **3**  **(15)** | **17**  **(102)** | **8**  **(56)** | **290**  **(4.82)** |
| Technological facilitators | | | | | | |  |  |  |
|  | **ICT (Information communication technology)** | | | | | | | | |
| 57. | **In the hospital, there is information communication technology infrastructure (e.g. Intranet, Extranet, PACS, and so on )** | **0**  **(0)** | **0**  **(0)** | **0**  **(0)** | **1**  **(4)** | **2**  **(10)** | **12**  **(72)** | **14**  **(98)** | **184**  **(6.34)** |
| 58. | **I believe that social media has a significant impact on knowledge sharing behaviours** | **2**  **(2)** | **2**  **(4)** | **2**  **(6)** | **5**  **(20)** | **12**  **(60)** | **3**  **(18)** | **3**  **(21)** | **131**  **(4.51)** |
| 59. | **There are technical support and maintenance groups available to address information communication technology related problems.** | **0**  **(0)** | **2**  **(4)** | **1**  **(3)** | **3**  **(12)** | **4**  **(20)** | **10**  **(60)** | **9**  **(63)** | **162**  **(5.58)** |
| 60. | **Employees in the hospital have the knowledge and skills to use information communication technology effectively.** | **1**  **(1)** | **1**  **(2)** | **1**  **(3)** | **6**  **(24)** | **6**  **(30)** | **8**  **(48)** | **6**  **(54)** | **162**  **(5.58)** |
| 61. | **Employees in the hospital use social information communication technology to communicate with each other.** | **0**  **(0)** | **2**  **(4)** | **1**  **(3)** | **8**  **(32)** | **8**  **(40)** | **5**  **(30)** | **5**  **(35)** | **144**  **(4.96)** |
| Total | | **3**  **(1)** | **7**  **(14)** | **5**  **(15)** | **22**  **(88)** | **30**  **(150)** | **38**  **(228)** | **37**  **(259)** | **755**  **(5.20)** |
|  | **Network** |  |  |  |  |  |  |  |  |
| 62. | **In the hospital, there is a high-speed network available.** | **1**  **(1)** | **1**  **(2)** | **0**  **(0)** | **7**  **(28)** | **5**  **(25)** | **7**  **(42)** | **8**  **(56)** | **154**  **(5.31)** |
| 63. | **I believe that an available network is vital in enabling knowledge sharing.** | **0**  **(0)** | **0**  **(0)** | **1**  **(3)** | **7**  **(28)** | **1**  **(5)** | **7**  **(42)** | **13**  **(91)** | **169**  **(5.82)** |
| Total | | **1**  **(1)** | **1**  **(2)** | **1**  **(3)** | **14**  **(56)** | **6**  **(30)** | **14**  **(84)** | **21**  **(147)** | **323**  **(5.56)** |
|  | **Digital library: It is an electronic recourse such as eBooks and databases, which are related to the medical background and published articles that support making decisions.** | | | | | | | |  |
| 64. | **I believe that digital libraries facilitate learning and therefore knowledge sharing.** | **0**  **(0)** | **0**  **(0)** | **1**  **(3)** | **6**  **(24)** | **4**  **(20)** | **10**  **(60)** | **8**  **(56)** | **163**  **(5.62)** |

**64. Feel Free to add any Comment regarding Knowledge sharing practices**

**Thank you**

**-The total 77**

**-Finished 29**

**-29 all working in the medical imaging department and radiotherapy.**
