# Supplementary material for: Evaluating Factors Affecting Knowledge Sharing Among Health Care Professionals in the Medical Imaging Departments of 2 Cancer Centers: Concurrent Mixed Methods Study
Source: JMIR Hum Factors. 2024 Nov 13;11:e53780. doi: 10.2196/53780 (PMC11602759; doi:10.2196/53780)
Supplement: Multimedia Appendix 5 [file humanfactors_v11i1e53780_app5.docx]

| **Multimedia Appendix 5: Definition of knowledge sharing in medical imaging departments based on the respondents’ views.** | | |
| --- | --- | --- |
| **Theme 1: Definition of knowledge sharing in the medical imaging department** | | |
| Participation | Work experience and job title | Participants quotes |
| - Participant (A)  - Participant (B)  - Participant (C)  - Participant (D)  Participant (E)  Participant (F)  - Participant (G)  -Participant (H)  -Participant (I)  -Participant (J)  -Participant (K)  -Participant (L)  -Participant (M) | 16 years as nuclear medicine technologist at KCCC  15 years as a nuclear medicine specialised doctor  11 years as a nuclear medicine specialised doctor.  6 yeas as a head of department of radiology.  17 years as a nurse partitioner  12 years as technologists, and 7 years as a chief of nuclear medicine technologists  14 years as a nurse in the radiology department  17 years a nuclear medicine technologist  16 years as radiologists  17 years as a nurse in charge  12 years as nurse  20 yeas as a radiologist, and 3 years as a head of radiologist  27 years in NHS | *“Distribute the information between our stuff for example through lectures”*  “*I believe that the knowledge that we gain during our year of experience has to be passed to other generations or even to other colleagues of the same age or even at an older age. So being able to be in a position where I am involved in some special. And another field the nuclear medicine and that leads to being one or two in the centre. This kind of knowledge has to be passed to other colleagues. And there are times where I might not be able to add more to that knowledge. Maybe other colleagues might propagate the knowledge to a much higher level or there are times that I might not be present in that place for any reason medical, social”*  *“In my opinion, knowledge sharing is very important amongst clinicians of various specialities and various field. Like for example, I'm in radiology, but definitely I would like to share my knowledge and also receive input. From all the other departments, especially with respect to since I'm working in Kuwait Cancer Centre for with respect to treating the patient. The teamwork in caring for the patient and treating the patient. So, I feel it's very important to share knowledge with the for example, nuclear medicine department or medical oncology radiation oncology surgical oncology. And basically, in all the various medical.*  *“As you are now I understand what, what do you mean? So, as I said, we have two parts. You know, especially we we've been in medical school, and we already have already a knowledge which is already there and what time and especially I came before I came for example in cases I've been in another hospital, I worked there in addition to attending conferences in addition to being through experience probably the rest of the stuff, they didn't go through. So to share the knowledge it will be either plan or through our meeting like we having a we used to have an everyday meeting and then we push we we probably we make it every Monday and Wednesday for the time until before you know Ramadan in April so and again according to that to the to the need I mean certain query was raised probably here you would like to you raise it to share it or it will be a planned as giving teaching or a pre-planned things. Checking the case and releasing the patient out of the scanner as much as you can and try to explain to the patient more about the procedure. Sometimes they just read the instruction they probably they don't. Put in mind that anything can happen so again, I've been through this so I shared it today and again next week. Probably something else will happen and we'd like to. And according to the situation”*  *“Knowledge sharing to me suggests how we disseminate information between the team. Urgent clinical information that needs addressing straight away like information that people have learned through doing a course and sharing information”*  *“Knowledge sharing regarding our job, is to share your knowledge, your information whatever, your experience with others especially the workers to have the good duty and good practice”*  *“That means that I have to share my own experience with all the stuff and you knew things. Honey, I learned I have to give to the new stuff, to the other stuff, and they have to share me also the new things that I don't”*  *“Any things in you that we can get it or any information that we get with the along with the patients or with the working like a technical part or a medical part if I get this information, I can share it with my colleague or sometimes we can share it with the patients according to the situation”*  *“As I know, it's like sharing your information. You're your knowledge. Whatever you have studied to other people to increase their knowledge so that you live them, know what you know and again it will be like sharing. You will get also what they you know”*  *“If we tell our knowledge to others, it will help, but that too. get to the knowledge to others and I can share my knowledge to others so it will be help with the patients and the staff also”*  *“Sharing the knowledge means share information. It a big help for the other people also. Like if we give the proper instructions through the patient. It will be good for the understanding of the patient, and they will cooperate”*  “*Sharing of the knowledge for the patients of this is on how we are, uh, interact with the patient on how we interact as well with our technologies and our colleagues with this one. So in case of for example that we have, we are like me as say for example as a senior and we have also as a junior. So, we are sharing our knowledge, not our the one that I knew for the for, for what I experienced for 20 years. I will share this one as well to my colleague who are coming. Who are new for us in the department in in our hospital? Which you know, we are cancer, cancer, patient cancer, hospital specialists in concert hospital. So how do we care of patients and how we treat a patient and how do we do the procedures in each patient as a technologist for the conservation?*  *“Sharing information and skills relevant to your work with the wider team”* |
